# Supplementary material for: Environmental Sampling Methods for Detection of Salmonella Infections in Laying Hens: A Systematic Review and Meta-Analysis
Source: Microorganisms. 2023 Aug 17;11(8):2100. doi: 10.3390/microorganisms11082100 (PMC10459801; doi:10.3390/microorganisms11082100)
Supplement: Supplementary file 1 [file microorganisms-11-02100-s001.zip › microorganisms-2517717-supplementary/SM_Table_S1.pdf]

Table S1. Overview of 114 studies identified for full text screening, indicating which one was included (1) for particular analysis and reasons for exclusions.

Author	Eligible for qualitative analysis Q1, Q2	Eligible for quantitative analysis Q3	Excluded	Reasons for exclusion for qualitative/quantitative analysis	Eligible for ISO method question Q4	Reason for exclusion ISO method question
Adesiyun et al., (2014)	1	0	0		1	
Agunos et al., (2021)	1	0	0		1	
Aimey et al., (2013)	1	1	0		1	
Al-Nakhli, Al-Ogaily, & Nassar, (1999)	0	0	1	Not only for laying hens		
Altekruse, Koehler, Hickman-Brenner, Tauxe, & Ferris, (1993)	0	0	1	Study on phages		
Andoh et al., (2016)	1	0	0		1	
Arnold, Carrique-Mas, & Davies, (2010)	1	1	0		1	
Arnold, Carrique-Mas, McLaren, & Davies, (2011)	1	1	0		1	
Arnold, Martelli, McLaren, & Davies, (2014a)	1	1	0		1	
Arnold, Martelli, McLaren, & Davies, (2014b)	1	1	0		0	No details described for culture
Ayachi, Alloui, Bennoune, & Kassah-Laouar, (2010)	0	0	1	No laying hens		
Bouzidi et al., (2012)	1	0	0		1	
Carli, Eygor, & Caner, (2001)	0	0	1	No environmental samples		
Carrique-Mas, Breslin, Snow, et al., (2008)	1	0	0		1	
Carrique-Mas, Breslin, Sayers, et al., (2008)	1	0	0		1	
Castellan et al., (2004)	1	0	0		1	
Charlton et al., (2005)	1	0	0		1	
Chemaly et al., (2009)	0	0	1	No environmental samples		
Chousalkar, Gole, Caraguel, & Rault, (2016)	1	0	0		1	
Clep, (2014)	0	0	1	No laying hens		

Crabb, Gilkerson, & Browning, (2019)	1	0	0		1	
Dailey, Niemeier, Elkhoraibi, Senties-Cue, & Pitesky, (2017)	0	0	1	Samples outside farm		
R. H. Davies et al., (1997)	0	0	1	Hatchery		
R. Davies & Breslin, (2001)	1	1	0		1	
R. H. Davies & M. F. Breslin, (2003)	1	1	0		1	
R. Davies, Liebana, & Breslin, (2003)	1	1	0		1	
R. Davies & M. Breslin, (2003)	1	1	0		1	
R. Davies & Breslin, (2004)	1	0	0		1	
Davison, Benson, & Eckroade, (1995)	1	0	0		1	
Davison, Benson, Henzler, & Eckroade, (1999)	1	0	0		1	
Denagamage et al., (2016)	1	0	0		1	
Denagamage, Jayarao, Wallner-Pendleton, Patterson, & Kariyawasam, (2017)	0	0	1	No original data		
Dewaele et al., (2012)	1	0	0		1	
Diker et al., (2020)	1	0	0		1	
Fagbamila et al., (2017)	1	0	0		1	
Fotariya, Brahmbhatt, Nayak, & Patel, (2014)	0	0	1	Study for virulence genes		
Garber, Smeltzer, Fedorka-Cray, Ladely, & Ferris, (2003)	1	0	0		1	
Garcia, Anderson, Guard, Gast, & Jones, (2021)	1	0	0		1	
Gast, Mitchell, & Holt, (2004)	0	0	1	Different aim: Comparison of different culture media		
Gole, Caraguel, Sexton, Fowler, & Chousalkar, (2014)	1	1	0		1	
Gole, Torok, Sexton, Caraguel, & Chousalkar, (2014)	1	1	0		1	
Gole et al., (2017)	1	1	0		1	

Haque et al., (2021)	1	0	0		1	
Hao, Li, Wang, Zhang, & Cao, (2013)	1	0	0		1	
Hardy et al., (2019)	0	0	1	Hatchery, backyard flock		
Hasan & Lafta, (2021)	1	0	0		1	
Henzler, Ebel, Sanders, Kradel, & Mason, (1994)	1	0	0		1	
Henzler & Opitz, (1992)	1	0	0		1	
Henzler, Kradel, & Sischo, (1998)	1	0	0		1	
Humbert, Carraminana, Lalande, & Salvat, (1997)	0	0	1	Study at experimental facility		
Huneau-Salaun et al., (2009)	1	0	0		1	
Im et al., (2015)	1	0	0		1	
F. T. Jones, Rives, & Carey, (1995)	1	0	0		1	
D. R. Jones, Anderson, & Guard, (2012)	1	0	0		1	
D. R. Jones et al., (2015)	1	0	0		1	
D. R. Jones et al., (2016)	1	0	0		1	
Jouy et al., (2005)	1	0	0		1	
Kahya, Eyigor, & Carl, (2013)	0	0	1	No laying hens		
Kinde et al., (1996)	1	0	0		1	
Kinde et al., (2004)	1	0	0		0	No details described for culture
Kinde et al., (2005)	1	0	0		1	
Kinde et al., (2015)	0	0	1	Spiking experiments		
Kingsbury, Thom, Erskine, Olsen, & Soboleva, (2019)	1	0	0		1	
Klinkenberg, Thomas, Artavia, & Bouma, (2011)	0	0	1	Lack of raw data		
Kottwitz et al., (2013)	0	0	1	Different aim: serotype Salmonella strains		
Koutsoumanis et al., (2019)	0	0	1	No original data		
Leon-Velarde et al., (2004)	1	0	0		1	

Li, Liu, Li, Xu, & Zheng, (2018)	1	1	0		1	
Lopašovskỳ et al., (2015)	1	0	0		0	No details described for culture
Mahe et al., (2008)	1	0	0		1	
Matsumoto, Miyama, & Murakami, (2001)	1	1	0		1	
McWhorter, Davos, & Chousalkar, (2015)	0	0	1	No original data		
McWhorter & Chousalkar, (2020)	1	0	0		1	
Moffatt et al., (2017)	0	0	1	No original data		
Moraes et al., (2016)	1	0	0		1	
Murakami, Horikawa, Ito, & Otsuki, (2001)	1	0	0		1	
Murase et al., (2001)	1	0	0		1	
Murase, Miyahara, Sato, Otsuki, & Holt, (2006)	0	0	1	Environmental samples not specified		
Mutalib, McDonough, Shin, Patten, & Lein, (1992)	1	0	0		1	
Pajic et al., (2015)	0	0	1	No environmental samples		
Pieskus, Kazeniauskas, Butrimaite-Ambrozeviciene, Stanevicius, & Mauricas, (2008)	1	0	0		1	
Pitesky, Charlton, Bland, & Rolfe, (2013)	1	0	0		0	No details described for culture
Poppe, Irwin, Forsberg, Clarke, & Oggel, (1991)	1	0	0		1	
Pulido-Landinez, Sanchez-Ingunza, Guard, & Nascimento, (2014)	0	0	1	No environmental samples		
Rodriguez, Fandino, Donado, Guzman, & Verjan, (2015)	1	0	0		1	
Salazar et al., (2019)	1	0	0		1	
Saravanan et al., (2015)	1	0	0		1	
Sasaki et al., (2012)	1	0	0		1	

Sasipreeyajan, Jerngklinchan, Koowatananukul, & Saitanu, (1996)	1	0	0		1	
Schulz et al., (2011)	1	0	0		1	
Sevilla-Navarro et al., (2018)	0	0	1	Different aim: study on Autophage		
P. Sharma et al., (2018)	1	0	0		1	
S. Sharma, Fowler, Pant, Singh, & Wilkins, (2021)	1	0	0		1	
Sheldon, Li, Payne, Santos, & Anderson, (2006)	1	0	0		1	
Shirota, Umali, Suzuki, & Katoh, (2012)	1	0	0		1	
Singh, Yadav, Tripathi, & Singh, (2013)	1	0	0		0	No details described for culture
Sivaramalingam, McEwen, Pearl, Ojkic, & Guerin, (2013)	0	0	1	No laying hens		
Snow et al., (2007)	1	0	0		1	
Sodagari et al., (2020)	1	0	0		1	
Soria et al., (2017)	1	1	0		1	
St. Amand, Cassis, King, & Christianson, (2017)	1	0	0		1	
Stocki et al., (2007)	1	0	0		0	No details described for culture
Sylejmani, Musliu, Ramadani, Sparagano, & Hamidi, (2016)	1	0	0		1	
Trawinska, Saba, Wdowiak, Ondrasovicova, & Nowakowicz-Debek, (2008)	0	0	1	No laying hens		
Van Hoorebeke et al., (2009)	1	1	0		1	
Van Hoorebeke et al., (2010)	1	1	0		1	
Wales, Breslin, & Davies, (2006a)	1	0	0		1	
Wales, Breslin, & Davies, (2006b)	1	1	0		1	

Wallner-Pendleton, Patterson, Kariyawasam, Trampel, & Denagamage, (2014)	1	0	0		1	
White, Schlosser, Benson, Maddox, & Hogue, (1997)	1	0	0		1	
Wilson, Chandry, Turner, Courtice, & Fegan, (2021)	1	0	0		0	No details described for culture
Yamane et al., (2000)	1	0	0		1	
Zhang, Yan, & Ryser, (2006)	1	0	0		1	
Zijpp, Mollenhorst, Berentsen, & Boer, (2006)	0	0	1	Discussion paper		

Adesiyun, A., Webb, L., Musai, L., Louison, B., Joseph, G., Stewart-Johnson, A., . . . Rodrigo, S. (2014). Survey of Salmonella contamination in chicken layer farms in three Caribbean countries. *Journal of Food Protection, 77*(9), 1471-1480. Retrieved from http://www.foodprotection.org

Agunos, A., Gow, S. P., Leger, D. F., Flockhart, L., Daignault, D., Desruisseau, A., . . . Reid-Smith, R. J. (2021). Antimicrobial resistance and recovery of Salmonella, Campylobacter, and Escherichia coli from chicken egg layer flocks in Canadian sentinel surveillance sites using 2 types of sample matrices. *Canadian Journal of Veterinary Research, 85*(1), 27-35. Retrieved from https://www.ingentaconnect.com/contentone/cvma/cjvr/2021/00000085/00000001/art00005;jsessionid=13qoofmniinq3.x-ic-live-02#expand/collapse

Aimey, V., Hunte, K., Whitehall, P., Sanford, B., Trotman, M., Delgado, A., . . . Hernandez, J. (2013). Prevalence of and examination of exposure factors for Salmonella on commercial egg-laying farms in Barbados. *Preventive Veterinary Medicine, 110*(3/4), 489-496. doi:http://dx.doi.org/10.1016/j.prevetmed.2012.12.009

Al-Nakhli, H. M., Al-Ogaily, Z. H., & Nassar, T. J. (1999). Representative Salmonella serovars isolated from poultry and poultry environments in Saudi Arabia. *Revue Scientifique et Technique - Office International des Epizooties, 18*(3), 700-709.

Altekruse, S., Koehler, J., Hickman-Brenner, F., Tauxe, R. V., & Ferris, K. (1993). A comparison of Salmonella enteritidis phage types from egg-associated outbreaks and implicated laying flocks. *Epidemiology and Infection, 110*(1), 17-22. doi:http://dx.doi.org/10.1017/S0950268800050639

Andoh, L. A., Dalsgaard, A., Obiri-Danso, K., Newman, M. J., Barco, L., & Olsen, J. E. (2016). Prevalence and antimicrobial resistance of Salmonella serovars isolated from poultry in Ghana. *Epidemiology and Infection, 144*(15), 3288-3299. doi:http://dx.doi.org/10.1017/S0950268816001126

Arnold, M. E., Carrique-Mas, J. J., & Davies, R. H. (2010). Sensitivity of environmental sampling methods for detecting Salmonella Enteritidis in commercial laying flocks relative to the within-flock prevalence. *Epidemiology and Infection, 138*(3), 330-339. doi:http://dx.doi.org/10.1017/S0950268809990598

Arnold, M. E., Carrique-Mas, J. J., McLaren, I., & Davies, R. H. (2011). A comparison of pooled and individual bird sampling for detection of Salmonella in commercial egg laying flocks. *Preventive Veterinary Medicine, 99*(2/4), 176-184. doi:http://dx.doi.org/10.1016/j.prevetmed.2010.12.007

Arnold, M. E., Martelli, F., McLaren, I., & Davies, R. H. (2014a). Estimation of the rate of egg contamination from Salmonella-infected chickens. *Zoonoses and Public Health, 61*(1), 18-27. doi:http://dx.doi.org/10.1111/zph.12038

Arnold, M. E., Martelli, F., McLaren, I., & Davies, R. H. (2014b). Estimation of the sensitivity of environmental sampling for detection of Salmonella in commercial layer flocks post-introduction of national control programmes. *Epidemiology and Infection, 142*(5), 1061-1069. doi:http://dx.doi.org/10.1017/S0950268813002173

Ayachi, A., Alloui, N., Bennoune, O., & Kassah-Laouar, A. (2010). Survey of Salmonella serovars in broilers and laying breeding reproducers in Eastern Algeria. *Journal of Infection in Developing Countries, 4*(2), 103-106. doi:10.3855/jidc.562

Bouzidi, N., Aoun, L., Zeghdoudi, M., Bensouilah, M., Elgroud, R., Oucief, I., . . . Millemann, Y. (2012). Salmonella contamination of laying-hen flocks in two regions of Algeria. *Food Research International, 45*(2), 897-904. doi:http://dx.doi.org/10.1016/j.foodres.2011.05.027

Carli, K. T., Eygor, A., & Caner, V. (2001). Prevalence of Salmonella serovars in chickens in Turkey. *Journal of Food Protection, 64*(11), 1832-1835.

Carrique-Mas, J. J., Breslin, M., Sayers, A. R., McLaren, I., Arnold, M., & Davies, R. (2008). Comparison of environmental sampling methods for detecting Salmonella in commercial laying flocks in the UK. *Letters in Applied Microbiology, 47*(6), 514-519. doi:http://dx.doi.org/10.1111/j.1472-765X.2008.02450.x

Carrique-Mas, J. J., Breslin, M., Snow, L., Arnold, M. E., Wales, A., McLaren, I., & Davies, R. H. (2008). Observations related to the Salmonella EU layer baseline survey in the United Kingdom: follow-up of positive flocks and sensitivity issues. *Epidemiology and Infection, 136*(11), 1537-1546. doi:http://dx.doi.org/10.1017/S095026880700012X

Castellan, D. M., Kinde, H., Kass, P. H., Cutler, G., Breitmeyer, R. E., Bell, D. D., . . . Kuney, D. R. (2004). Descriptive study of California egg layer premises and analysis of risk factors for Salmonella enterica serotype enteritidis as characterized by manure drag swabs. *Avian Diseases, 48*(3), 550-561. doi:http://dx.doi.org/10.1637/7159-012804R1

Charlton, B. R., Walker, R. L., Kinde, H., Bauer, C. R., Channing-Santiago, S. E., & Farver, T. B. (2005). Comparison of a Salmonella Enteritidis-specific polymerase chain reaction assay to delayed secondary enrichment culture for the detection of Salmonella Enteritidis in environmental drag swab samples. *Avian Diseases, 49*(3), 418-422. doi:http://dx.doi.org/10.1637/7283-092404R1.1

Chemaly, M., Huneau-Salaun, A., Labbe, A., Houdayer, C., Petetin, I., & Fravalo, P. (2009). Isolation of Salmonella enterica in laying-hen flocks and assessment of eggshell contamination in France. *Journal of Food Protection, 72*(10), 2071-2077. Retrieved from http://www.foodprotection.org

Chousalkar, K., Gole, V., Caraguel, C., & Rault, J. L. (2016). Chasing Salmonella Typhimurium in free range egg production system. *Veterinary Microbiology, 192*, 67-72. doi:http://dx.doi.org/10.1016/j.vetmic.2016.06.013

Clep, R. (2014). Frequency of Salmonella spp. relevant serovars isolated during 2009-2012 from laying hens flocks. *Lucrari Stiintifice - Universitatea de Stiinte Agricole a Banatului Timisoara, Medicina Veterinara, 47*(3), 26-30.

Crabb, H. K., Gilkerson, J. R., & Browning, G. F. (2019). Does only the age of the hen matter in Salmonella enterica contamination of eggs? *Food Microbiology, 77*, 1-9. doi:http://dx.doi.org/10.1016/j.fm.2018.08.006

Dailey, N., Niemeier, D., Elkhoraibi, C., Senties-Cue, C. G., & Pitesky, M. (2017). Descriptive survey and Salmonella surveillance of pastured poultry layer farms in California. *Poultry Science, 96*(4), 957-965. Retrieved from https://academic.oup.com/ps/article/96/4/957/2282770/Descriptive-survey-and-Salmonella-surveillance-of

Davies, R., & Breslin, M. (2001). Environmental contamination and detection of Salmonella enterica serovar enteritidis in laying flocks. *Veterinary Record, 149*(23), 699-704.

Davies, R., & Breslin, M. (2003). Effects of vaccination and other preventive methods for Salmonella Enteritidis on commercial laying chicken farms. *Veterinary Record, 153*(22), 673-677.

Davies, R., & Breslin, M. (2004). Observations on Salmonella contamination of eggs from infected commercial laying flocks where vaccination for Salmonella enterica serovar Enteritidis had been used. *Avian Pathology, 33*(2), 133-144. doi:http://dx.doi.org/10.1080/03079450310001652040

Davies, R., Liebana, E., & Breslin, M. (2003). Investigation of the distribution and control of Salmonella enterica serovar Enteritidis PT6 in layer breeding and egg production. *Avian Pathology, 32*(3), 227-237. doi:http://dx.doi.org/10.1080/0307945031000097822

Davies, R. H., & Breslin, M. F. (2003). Observations on the distribution and persistence of Salmonella enterica serovar Enteritidis phage type 29 on a cage layer farm before and after the use of competitive exclusion treatment. *British Poultry Science, 44*(4), 551-557. doi:http://dx.doi.org/10.1080/00071660310001618307

Davies, R. H., Nicholas, R. A. J., McLaren, I. M., Corkish, J. D., Lanning, D. G., & Wray, C. (1997). Bacteriological and serological investigation of persistent Salmonella enteritidis infection in an integrated poultry organisation. *Veterinary Microbiology, 58*(2/4), 277-293. doi:http://dx.doi.org/10.1016/S0378-1135(97)00157-0

Davison, S., Benson, C. E., & Eckroade, R. J. (1995). Comparison of environmental monitoring protocols for the detection of Salmonella in poultry houses. *Avian Diseases, 39*(3), 475-479. doi:http://dx.doi.org/10.2307/1591798

Davison, S., Benson, C. E., Henzler, D. J., & Eckroade, R. J. (1999). Field observations with Salmonella enteritidis bacterins. *Avian Diseases, 43*(4), 664-669. doi:http://dx.doi.org/10.2307/1592735

Denagamage, T. N., Jayarao, B. M., Wallner-Pendleton, E., Patterson, P. H., & Kariyawasam, S. (2017). A retrospective study of Salmonella Enteritidis isolated from commercial layer flocks. *Avian Diseases, 61*(3), 330-334. doi:http://dx.doi.org/10.1637/11590-011817-RegR

Denagamage, T. N., Patterson, P., Wallner-Pendleton, E., Trampel, D., Shariat, N., Dudley, E. G., . . . Subhashinie, K. (2016). Longitudinal monitoring of successive commercial layer flocks for Salmonella enterica serovar Enteritidis. *Foodborne Pathogens and Disease, 13*(11), 618-625. Retrieved from http://online.liebertpub.com/fpd

Dewaele, I., Meirhaeghe, H. v., Rasschaert, G., Vanrobaeys, M., Graef, E. d., Herman, L., . . . Reu, K. d. (2012). Persistent Salmonella Enteritidis environmental contamination on layer farms in the context of an implemented national control program with obligatory vaccination. *Poultry Science, 91*(2), 282-291. doi:http://dx.doi.org/10.3382/ps.2011-01673

Diker, K. S., Goncuoglu, M., Sahin, G., Akan, M., Gurcan, I. S., Mustak, H. K., . . . iftci, M. M. (2020). Base study for the establishment of national Salmonella control program in hatching farms and table eggs in Turkey. *Turkish Journal of Veterinary & Animal Sciences, 44*(2), 343-349. doi:http://dx.doi.org/10.3906/vet-1908-2

Fagbamila, I. O., Barco, L., Mancin, M., Kwaga, J., Ngulukun, S. S., Zavagnin, P., . . . Muhammad, M. (2017). Salmonella serovars and their distribution in Nigerian commercial chicken layer farms. *PLoS ONE, 12*(3), e0173097. doi:http://dx.doi.org/10.1371/journal.pone.0173097

Fotariya, M. H., Brahmbhatt, M. N., Nayak, J. B., & Patel, B. D. (2014). Virulence genes characterization of Salmonella serovars isolated from eggs and poultry house environment. *Veterinary Practitioner, 15*(1), 86-88. Retrieved from http://www.vetpract.in/Archives/June-2014/VP-15-1--JUNE-2014/

Garber, L., Smeltzer, M., Fedorka-Cray, P., Ladely, S., & Ferris, K. (2003). Salmonella enterica serotype enteritidis in table egg layer house environments and in mice in U.S. layer houses and associated risk factors. *Avian Diseases, 47*(1), 134-142. doi:http://dx.doi.org/10.1637/0005-2086(2003)047[0134:SESEIT]2.0.CO;2

Garcia, J. S., Anderson, K. E., Guard, J. Y., Gast, R. K., & Jones, D. R. (2021). Impact of organic dairy cattle manure on environmental and egg microbiology of organic free-range laying hens. *Journal of Applied Poultry Research, 30*(4). doi:http://dx.doi.org/10.1016/j.japr.2021.100189

Gast, R. K., Mitchell, B. W., & Holt, P. S. (2004). Evaluation of culture media for detecting airborne Salmonella enteritidis collected with an electrostatic sampling device from the environment of experimentally infected laying hens. *Poult Sci, 83*(7), 1106-1111. doi:10.1093/ps/83.7.1106

Gole, V. C., Caraguel, C. G. B., Sexton, M., Fowler, C., & Chousalkar, K. K. (2014). Shedding of Salmonella in single age caged commercial layer flock at an early stage of lay. *International Journal of Food Microbiology, 189*, 61-66. doi:http://dx.doi.org/10.1016/j.ijfoodmicro.2014.07.030

Gole, V. C., Torok, V., Sexton, M., Caraguel, C. G. B., & Chousalkar, K. K. (2014). Association between indoor environmental contamination by Salmonella enterica and contamination of eggs on layer farms. *Journal of Clinical Microbiology, 52*(9), 3250-3258. doi:http://dx.doi.org/10.1128/JCM.00816-14

Gole, V. C., Woodhouse, R., Caraguel, C., Moyle, T., Rault, J. L., Sexton, M., & Chousalkar, K. (2017). Dynamics of Salmonella shedding and welfare of hens in free-range egg production systems. *Applied and Environmental Microbiology, 83*(5), e03313. doi:http://dx.doi.org/10.1128/aem.03313-16

Hao, X. X., Li, B. M., Wang, C. Y., Zhang, Q., & Cao, W. (2013). Application of slightly acidic electrolyzed water for inactivating microbes in a layer breeding house. *Poultry Science, 92*(10), 2560-2566. doi:http://dx.doi.org/10.3382/ps.2013-03117

Haque, A. K. M. Z., Akter, M. R., Islam, S. K. S., Jahangir, A., Neogi, S. B., Yamasaki, S., & Kabir, S. M. L. (2021). Salmonella gallinarum in small-scale commercial layer flocks: occurrence, molecular diversity and antibiogram. *Veterinary Sciences, 8*(5). doi:http://dx.doi.org/10.3390/vetsci8050071

Hardy, M. C., Robertson, S. A., Sidge, J., Signs, K., Stobierski, M. G., Jones, K., . . . Hise, K. B. (2019). Environmental investigation of a multistate salmonellosis outbreak linked to live backyard

poultry from a mail-order hatchery - Michigan, 2018. *Morbidity and Mortality Weekly Report, 67*(51/52), 1430-1431. doi:http://dx.doi.org/10.15585/mmwr.mm675152a5

Hasan, T. O., & Lafta, I. J. (2021). Identification and antimicrobial susceptibility profiles of Salmonella spp. isolated from chicken flocks and their feed and water in karbala, Iraq. *Indian Journal of Ecology, 48*(5), 1542-1550. Retrieved from http://indianecologicalsociety.com/society/wp-content/themes/ecology/volume_pdfs/1637035810.pdf

Henzler, D. J., Ebel, E., Sanders, J., Kradel, D., & Mason, J. (1994). Salmonella enteritidis in eggs from commercial chicken layer flocks implicated in human outbreaks. *Avian Diseases, 38*(1), 37-43. doi:http://dx.doi.org/10.2307/1591834

Henzler, D. J., Kradel, D. C., & Sischo, W. M. (1998). Management and environmental risk factors for Salmonella enteritidis contamination of eggs. *American Journal of Veterinary Research, 59*(7), 824-829. Retrieved from https://www.scopus.com/inward/record.uri?eid=2-s2.0-0032109450&partnerID=40&md5=d0a6b15b9fbae43414fe9a57243f10b2

Henzler, D. J., & Opitz, H. M. (1992). The role of mice in the epizootiology of Salmonella enteritidis infection on chicken layer farms. *Avian Diseases, 36*(3), 625-631. doi:http://dx.doi.org/10.2307/1591757

Humbert, F., Carraminana, J. J., Lalande, F., & Salvat, G. (1997). Bacteriological monitoring of Salmonella enteritidis carrier birds after decontamination using enrofloxacin, competitive exclusion and movement of birds. *Veterinary Record, 141*(12), 297-299.

Huneau-Salaun, A., Chemaly, M., Bouquin, S. l., Lalande, F., Petetin, I., Rouxel, S., . . . Rose, N. (2009). Risk factors for Salmonella enterica subsp. enterica contamination in 519 French laying hen flocks at the end of the laying period. *Preventive Veterinary Medicine, 89*(1/2), 51-58. doi:http://dx.doi.org/10.1016/j.prevetmed.2009.01.006

Im, M., Jeong, S., Kwon, Y., Jeong, O., Kang, M., & Lee, Y. (2015). Prevalence and characteristics of Salmonella spp. isolated from commercial layer farms in Korea. *Poultry Science, 94*(7), 1691-1698. Retrieved from http://ps.oxfordjournals.org/content/94/7/1691.full

Jones, D. R., Anderson, K. E., & Guard, J. Y. (2012). Prevalence of coliforms, Salmonella, Listeria, and Campylobacter associated with eggs and the environment of conventional cage and free-range egg production. *Poultry Science, 91*(5), 1195-1202. doi:http://dx.doi.org/10.3382/ps.2011-01795

Jones, D. R., Cox, N. A., Guard, J., Fedorka-Cray, P. J., Buhr, R. J., Gast, R. K., . . . Makagon, M. M. (2015). Microbiological impact of three commercial laying hen housing systems. *Poultry Science, 94*(3), 544-551. doi:http://dx.doi.org/10.3382/ps/peu010

Jones, D. R., Guard, J., Gast, R. K., Buhr, R. J., Fedorka-Cray, P. J., Abdo, Z., . . . Karcher, D. M. (2016). Influence of commercial laying hen housing systems on the incidence and identification of Salmonella and Campylobacter. *Poultry Science, 95*(5), 1116-1124. doi:http://dx.doi.org/10.3382/ps/pew036

Jones, F. T., Rives, D. V., & Carey, J. B. (1995). Salmonella contamination in commercial eggs and an egg production facility. *Poultry Science, 74*(4), 753-757. doi:http://dx.doi.org/10.3382/ps.0740753

Jouy, E., Proux, K., Humbert, F., Rose, V., Lalande, F., Houdayer, C., . . . Salvat, G. (2005). Evaluation of a French ELISA for the detection of Salmonella Enteritidis and Salmonella Typhimurium in flocks of laying and breeding hens. *Preventive Veterinary Medicine, 71*(1/2), 91-103. doi:http://dx.doi.org/10.1016/j.prevetmed.2005.06.003

Kahya, S., Eyigor, A., & Carl, K. T. (2013). Comparison of different sample types for Salmonella detection from chicken layer breeder flocks. *Veteriner Fakultesi Dergisi, Uludag Universitesi, 32*(2), 19-24. Retrieved from http://veteriner.uludag.edu.tr

Kinde, H., Castellan, D. M., Kass, P. H., Ardans, A., Cutler, G., Breitmeyer, R. E., . . . Kuney, D. R. (2004). The occurrence and distribution of Salmonella enteritidis and other serovars on California egg laying premises: a comparison of two sampling methods and two culturing techniques. *Avian Diseases, 48*(3), 590-594. doi:http://dx.doi.org/10.1637/7165-021104R

Kinde, H., Castellan, D. M., Kerr, D., Campbell, J., Breitmeyer, R., & Ardans, A. (2005). Longitudinal monitoring of two commercial layer flocks and their environments for Salmonella enterica

serovar Enteritidis and other salmonellae. *Avian Diseases, 49*(2), 189-194. doi:http://dx.doi.org/10.1637/7228-062704R

Kinde, H., Goodluck, H. A., Pitesky, M., Friend, T. D., Campbell, J. A., & Hill, A. E. (2015). Validation of single and pooled manure drag swabs for the detection of Salmonella serovar Enteritidis in commercial poultry houses. *Avian Diseases, 59*(4), 548-553. doi:http://dx.doi.org/10.1637/11224-063015-ResNote.1

Kinde, H., Read, D. H., Chin, R. P., Bickford, A. A., Walker, R. L., Ardans, A., . . . Gardner, I. A. (1996). Salmonella enteritidis, phage type 4 infection in a commercial layer flock in southern California: bacteriologic and epidemiologic findings. *Avian Diseases, 40*(3), 665-671. doi:http://dx.doi.org/10.2307/1592279

Kingsbury, J. M., Thom, K., Erskine, H., Olsen, L., & Soboleva, T. (2019). Prevalence and genetic analysis of Salmonella enterica from a cross-sectional survey of the New Zealand egg production environment. *Journal of Food Protection, 82*(12), 2201-2214. Retrieved from https://jfoodprotection.org/doi/abs/10.4315/0362-028X.JFP-19-159

Klinkenberg, D., Thomas, E., Artavia, F. F. C., & Bouma, A. (2011). Salmonella enteritidis surveillance by egg immunology: impact of the sampling scheme on the release of contaminated table eggs. *Risk Analysis, 31*(8), 1260-1270. doi:http://dx.doi.org/10.1111/j.1539-6924.2011.01582.x

Kottwitz, L. B. M., Back, A., Leao, J. A., Frausto, H. S. E., Magnani, M., & Oliveira, T. C. R. M. d. (2013). Decline of Salmonella Enteritidis in poultry. *British Food Journal, 115*(11), 1541-1546. doi:http://dx.doi.org/10.1108/BFJ-04-2012-0086

Koutsoumanis, K., Allende, A., Alvarez-Ordonez, A., Bolton, D., Bover-Cid, S., Chemaly, M., . . . Davies, R. (2019). Salmonella control in poultry flocks and its public health impact. *EFSA Journal, 17*(2), e05596. Retrieved from https://efsa.onlinelibrary.wiley.com/doi/full/10.2903/j.efsa.2019.5596

Leon-Velarde, C. G., Cai, H. Y., Larkin, C., Bell-Rogers, P., Stevens, R. W. C., & Odumeru, J. A. (2004). Evaluation of methods for the identification of Salmonella enterica serotype Typhimurium [S. typhimurium] DT104 from poultry environmental samples. *Journal of Microbiological Methods, 58*(1), 79-86. doi:http://dx.doi.org/10.1016/j.mimet.2004.03.005

Li, X., Liu, L., Li, Q., Xu, G., & Zheng, J. (2018). Salmonella contamination in layer farms in China: detection and genetic analysis. *Journal of Poultry Science, 55*(1), 1-9. doi:http://dx.doi.org/10.2141/jpsa.0160144

Lopašovskỳ, L., Zeleňáková, L., Fikselová, M., Bobková, A., Kunová, S., Bobko, M., & Šnirc, M. (2015). The prevalence of Salmonella infections in laying hen flocks producing eggs and their impact on the public health. *Potravinarstvo, 9*(1), 228-236. doi:10.5219/461

Mahe, A., Bougeard, S., Huneau-Salaun, A., Bouquin, S. l., Petetin, I., Rouxel, S., . . . Rose, N. (2008). Bayesian estimation of flock-level sensitivity of detection of Salmonella spp., Enteritidis and Typhimurium according to the sampling procedure in French laying-hen houses. *Preventive Veterinary Medicine, 84*(1/2), 11-26. doi:http://dx.doi.org/10.1016/j.prevetmed.2007.10.003

Matsumoto, A., Miyama, M., & Murakami, S. (2001). Comparison of Salmonella isolation rates in different types of egg-layer hen houses in Chiba, Japan. *Avian Diseases, 45*(1), 195-200. doi:http://dx.doi.org/10.2307/1593028

McWhorter, A. R., & Chousalkar, K. K. (2020). Salmonella on Australian cage egg farms: observations from hatching to end of lay. *Food Microbiology, 87*, 103384. doi:http://dx.doi.org/10.1016/j.fm.2019.103384

McWhorter, A. R., Davos, D., & Chousalkar, K. K. (2015). Pathogenicity of Salmonella strains isolated from egg shells and the layer farm environment in Australia. *Applied and Environmental Microbiology, 81*(1), 405-414. doi:http://dx.doi.org/10.1128/AEM.02931-14

Moffatt, C. R. M., Musto, J., Pingault, N., Combs, B., Miller, M., Stafford, R., . . . Kirk, M. D. (2017). Recovery of Salmonella enterica from Australian layer and processing environments following outbreaks linked to eggs. *Foodborne Pathogens and Disease, 14*(8), 478-482. doi:http://dx.doi.org/10.1089/fpd.2016.2268

Moraes, D. M. C., Andrade, M. A., Duarte, S. C., Bastos, T. S. A., Arnhold, E., Jayme, V. d. S., & Nunes, I. A. (2016). Phenotypic and molecular detection of Salmonella sp. on growing, rearing and production phases in a commercial group of laying hens. *Pesquisa Veterinaria Brasileira, 36*(6), 503-508. Retrieved from http://130.211.64.33/pdf_artigos/07-06-2016_18-18Vet%202133_4102hH%20LD.pdf

Murakami, K., Horikawa, K., Ito, T., & Otsuki, K. (2001). Environmental survey of salmonella and comparison of genotypic character with human isolates in Western Japan. *Epidemiology and Infection, 126*(2), 159-171. doi:http://dx.doi.org/10.1017/S0950268801005283

Murase, T., Miyahara, S., Sato, T., Otsuki, K., & Holt, P. S. (2006). Isolation of Salmonella organisms from commercial layer houses where the flocks were molted with a wheat bran diet. *Journal of Applied Poultry Research, 15*(1), 116-121. doi:http://dx.doi.org/10.1093/japr/15.1.116

Murase, T., Senjyu, K., Maeda, T., Tanaka, M., Sakae, H., Matsumoto, Y., . . . Otsuki, K. (2001). Monitoring of chicken houses and an attached egg-processing facility in a laying farm for Salmonella contamination between 1994 and 1998. *Journal of Food Protection, 64*(12), 1912-1916.

Mutalib, A., McDonough, P., Shin, S., Patten, V., & Lein, D. (1992). Salmonella enteritidis in commercial layer farms in New York state; environmental survey results and significance of available monitoring tests. *Journal of Veterinary Diagnostic Investigation, 4*(4), 416-418.

Pajic, M., Todorovic, D., Velhner, M., Milanov, D., Polacek, V., uric, S., & Stojanov, I. (2015). The epizootiological importance of Salmonella spp. isolated in various aspects of poultry production in the Southern Backa and Srem region. *Arhiv Veterinarske Medicine, 8*(1), 67-76. Retrieved from http://niv.ns.ac.rs/wp-content/uploads/2015/12/7.Pajic.pdf

Pieskus, J., Kazeniauskas, E., Butrimaite-Ambrozeviciene, C., Stanevicius, Z., & Mauricas, M. (2008). Salmonella incidence in broiler and laying hens with the different housing systems. *Journal of Poultry Science, 45*(3), 227-231. doi:http://dx.doi.org/10.2141/jpsa.45.227

Pitesky, M., Charlton, B., Bland, M., & Rolfe, D. (2013). Surveillance of Salmonella Enteritidis in layer houses: a retrospective comparison of the Food and Drug Administration's Egg Safety Rule (2010-2011) and the California Egg Quality Assurance Program (2007-2011). *Avian Diseases, 57*(1), 51-56. doi:http://dx.doi.org/10.1637/10281-061312-Reg.1

Poppe, C., Irwin, R. J., Forsberg, C. M., Clarke, R. C., & Oggel, J. (1991). The prevalence of Salmonella enteritidis and other Salmonella spp. among Canadian registered commercial layer flocks. *Epidemiology and Infection, 100*(2), 259-270. doi:http://dx.doi.org/10.1017/S0950268800048408

Pulido-Landinez, M., Sanchez-Ingunza, R., Guard, J., & Nascimento, V. P. d. (2014). Presence of Salmonella Enteritidis and Salmonella Gallinarum in commercial laying hens diagnosed with fowl typhoid disease in Colombia. *Avian Diseases, 58*(1), 165-170. doi:http://dx.doi.org/10.1637/10598-062613-Case.1

Rodriguez, R., Fandino, C., Donado, P., Guzman, L., & Verjan, N. (2015). Characterization of Salmonella from commercial egg-laying hen farms in a central region of Colombia. *Avian Diseases, 59*(1), 57-63. doi:http://dx.doi.org/10.1637/10873-052714-Reg

Salazar, G. A., Guerrero-López, R., Lalaleo, L., Avilés-Esquivel, D., Vinueza-Burgos, C., & Calero-Cáceres, W. (2019). Presence and diversity of Salmonella isolated from layer farms in central Ecuador. *F1000Research, 8*. doi:10.12688/f1000research.18233.2

Saravanan, S., Purushothaman, V., Murthy, T. R., Sukumar, K., Srinivasan, P., Gowthaman, V., . . . Kuchipudi, S. V. (2015). Molecular Epidemiology of Nontyphoidal Salmonella in Poultry and Poultry Products in India: Implications for Human Health. *Indian J Microbiol, 55*(3), 319-326. doi:10.1007/s12088-015-0530-z

Sasaki, Y., Murakami, M., Maruyama, N., Tsujiyama, Y., Kusukawa, M., Asai, T., & Yamada, Y. (2012). Risk factors for Salmonella prevalence in laying-hen farms in Japan. *Epidemiology and Infection, 140*(6), 982-990. doi:http://dx.doi.org/10.1017/S0950268811001506

Sasipreeyajan, J., Jerngklinchan, J., Koowatananukul, C., & Saitanu, K. (1996). Prevalence of salmonellae in broiler, layer and breeder flocks in Thailand. *Tropical Animal Health and Production, 28*(2), 174-180. doi:http://dx.doi.org/10.1007/BF03030841

Schulz, J., Hoorebeke, S. v., Hald, B., Hartung, J., Immerseel, F. v., Radtke, I., . . . Dewulf, J. (2011). The dynamics of Salmonella occurrence in commercial laying hen flocks throughout a laying period. *Avian Pathology, 40*(3), 243-248. doi:http://dx.doi.org/10.1080/03079457.2010.544290

Sevilla-Navarro, S., Marin, C., Cortes, V., Garcia, C., Vega, S., & Catala-Gregori, P. (2018). Autophage as a control measure for Salmonella in laying hens. *Poultry Science, 97*(12), 4367-4373. Retrieved from https://academic.oup.com/ps/article-abstract/97/12/4367/5049877

Sharma, P., Caraguel, C., Sexton, M., McWhorter, A., Underwood, G., Holden, K., & Chousalkar, K. (2018). Shedding of Salmonella Typhimurium in vaccinated and unvaccinated hens during early lay in field conditions: a randomised controlled trial. *BMC Microbiology, 18*(1), 78. doi:10.1186/s12866-018-1201-0

Sharma, S., Fowler, P. D., Pant, D. K., Singh, S., & Wilkins, M. J. (2021). Prevalence of non-typhoidal Salmonella and risk factors on poultry farms in Chitwan, Nepal. *Veterinary World, 14*(2), 426-436. doi:http://dx.doi.org/10.14202/vetworld.2021.426-436

Sheldon, B. W., Li, X., Payne, J. B., Santos, F. B. O., & Anderson, K. E. (2006). Surveillance of Salmonella populations, prevalence and serotype diversity in layer feces as a function of production cycle and molting. *EPC 2006 - 12th European Poultry Conference, Verona, Italy, 10-14 September, 2006*, paper-293.

Shirota, K., Umali, D. V., Suzuki, T., & Katoh, H. (2012). Epizootiologic role of feeds in the epidemiology of Salmonella Senftenberg contamination in commercial layer farms in eastern Japan. *Avian Diseases, 56*(3), 516-520. doi:http://dx.doi.org/10.1637/9964-101611-Reg.1

Singh, R., Yadav, A. S., Tripathi, V., & Singh, R. P. (2013). Antimicrobial resistance profile of Salmonella present in poultry and poultry environment in north India. *Food Control, 33*(2), 545-548. doi:https://doi.org/10.1016/j.foodcont.2013.03.041

Sivaramalingam, T., McEwen, S. A., Pearl, D. L., Ojkic, D., & Guerin, M. T. (2013). A temporal study of Salmonella serovars from environmental samples from poultry breeder flocks in Ontario between 1998 and 2008. *Canadian Journal of Veterinary Research, 77*(1), 1-11. Retrieved from http://www.ingentaconnect.com/content/cvma/cjvr/2013/00000077/00000001/art00001

Snow, L. C., Davies, R. H., Christiansen, K. H., Carrique-Mas, J. J., Wales, A. D., O'Connor, J. L., . . . Evans, S. J. (2007). Survey of the prevalence of Salmonella species on commercial laying farms in the United Kingdom. *Veterinary Record, 161*(14), 471-476. Retrieved from http://veterinaryrecord.bvapublications.com/archive/

Sodagari, H. R., Habib, I., Whiddon, S., Wang, P., Mohammed, A. B., Robertson, I., & Goodchild, S. (2020). Occurrence and characterization of Salmonella isolated from table egg layer farming environments in Western Australia and insights into biosecurity and egg handling practices. *Pathogens, 9*(1). doi:10.3390/pathogens9010056

Soria, M. C., Soria, M. A., Bueno, D. J., Godano, E. I., Gomez, S. C., ViaButron, I. A., . . . Roge, A. D. (2017). Salmonella spp. contamination in commercial layer hen farms using different types of samples and detection methods. *Poultry Science, 96*(8), 2820-2830. doi:http://dx.doi.org/10.3382/ps/pex053

St. Amand, J. A., Cassis, R., King, R. K., & Christianson, C. B. A. (2017). Prevalence of Salmonella spp. in environmental samples from table egg barns in Alberta. *Avian Pathology, 46*(6), 594-601. doi:http://dx.doi.org/10.1080/03079457.2017.1311989

Stocki, S. L., Annett, C. B., Sibley, C. D., McLaws, M., Checkley, S. L., Singh, N., . . . White, A. P. (2007). Persistence of Salmonella on egg conveyor belts is dependent on the belt type but not on the rdar morphotype. *Poultry Science, 86*(11), 2375-2383. doi:http://dx.doi.org/10.3382/ps.2007-00121

Sylejmani, D., Musliu, A., Ramadani, N., Sparagano, O., & Hamidi, A. (2016). Associations between the level of biosecurity and occurrence of Dermanyssus gallinae and Salmonella spp. in layer farms. *Avian Diseases, 60*(2), 454-459. Retrieved from http://www.bioone.org/loi/avdi

Trawinska, B., Saba, L., Wdowiak, L., Ondrasovicova, O., & Nowakowicz-Debek, B. (2008). Evaluation of Salmonella rod incidence in poultry in the Lublin Province over the years 2001-2005. *Annals*

of Agricultural and Environmental Medicine, 15(1), 131-134. Retrieved from http://www.aaem.pl/pdf/15131.pdf

Van Hoorebeke, S., Van Immerseel, F., De Vylder, J., Ducatelle, R., Haesebrouck, F., Pasmans, F., . . . Dewulf, J. (2009). Faecal sampling underestimates the actual prevalence of Salmonella in laying hen flocks. *Zoonoses Public Health, 56*(8), 471-476. doi:10.1111/j.1863-2378.2008.01211.x

Van Hoorebeke, S., Van Immerseel, F., Schulz, J., Hartung, J., Harisberger, M., Barco, L., . . . Dewulf, J. (2010). Determination of the within and between flock prevalence and identification of risk factors for Salmonella infections in laying hen flocks housed in conventional and alternative systems. *Prev Vet Med, 94*(1-2), 94-100. doi:10.1016/j.prevetmed.2009.11.022

Wales, A., Breslin, M., & Davies, R. (2006a). Assessment of cleaning and disinfection in Salmonella-contaminated poultry layer houses using qualitative and semi-quantitative culture techniques. *Veterinary Microbiology, 116*(4), 283-293. doi:10.1016/j.vetmic.2006.04.026

Wales, A., Breslin, M., & Davies, R. (2006b). Semiquantitative assessment of the distribution of Salmonella in the environment of caged layer flocks. *Journal of Applied Microbiology, 101*(2), 309-318. doi:http://dx.doi.org/10.1111/j.1365-2672.2006.02916.x

Wallner-Pendleton, E. A., Patterson, P. H., Kariyawasam, S., Trampel, D. W., & Denagamage, T. (2014). On-farm risk factors for Salmonella Enteritidis contamination. *Journal of Applied Poultry Research, 23*(2), 345-352. doi:http://dx.doi.org/10.3382/japr.2014-00943

White, P. L., Schlosser, W., Benson, C. E., Maddox, C., & Hogue, A. (1997). Environmental survey by manure drag sampling for Salmonella enteritidis in chicken layer houses. *Journal of Food Protection, 60*(10), 1189-1193.

Wilson, A., Chandry, P. S., Turner, M. S., Courtice, J. M., & Fegan, N. (2021). Comparison between cage and free-range egg production on microbial composition, diversity and the presence of Salmonella enterica. *Food Microbiology, 97*. doi:http://dx.doi.org/10.1016/j.fm.2021.103754

Yamane, Y., Leonard, J. D., Kobatake, R., Awamura, N., Toyota, Y., Ohta, H., . . . Inoue, T. (2000). A case study on Salmonella enteritidis (SE) origin at three egg-laying farms and its control with a S. enteritidis bacterin. *Avian Diseases, 44*(3), 519-526. doi:http://dx.doi.org/10.2307/1593090

Zhang, L., Yan, Z., & Ryser, E. T. (2006). Comparison of the reveal test, the U.S. Food and Drug Administration culture method, and selective media for recovery of Salmonella enteritidis from commercial egg layer flock environments. *Journal of Food Protection, 69*(11), 2766-2769. Retrieved from http://www.foodprotection.org

Zijpp, A. J. v. d., Mollenhorst, H., Berentsen, P. B. M., & Boer, I. J. M. d. (2006). Alternatives for the battery cage system: a comparison of economic, environmental and societal performance. *EPC 2006 - 12th European Poultry Conference, Verona, Italy, 10-14 September, 2006*, paper-206.
